# A User’s Guide to Cell-Free Protein Synthesis

**DOI:** 10.3390/mps2010024

**Published:** 2019-03-12

**Authors:** Nicole E. Gregorio, Max Z. Levine, Javin P. Oza

**Affiliations:** 1 Center for Applications in Biotechnology, California Polytechnic State University, San Luis Obispo, CA 93407, USA; negregor@calpoly.edu (N.E.G.); mzlevine@calpoly.edu (M.Z.L.); 2 Department of Chemistry and Biochemistry, California Polytechnic State University, San Luis Obispo, CA 93407, USA; 3 Department of Biological Sciences, California Polytechnic State University, San Luis Obispo, CA 93407, USA

**Keywords:** cell-free protein synthesis (CFPS), in vitro transcription-translation (TX-TL), cell-free protein expression (CFPE), in vitro protein synthesis, cell-free synthetic biology, cell-free metabolic engineering (CFME)

## Abstract

Cell-free protein synthesis (CFPS) is a platform technology that provides new opportunities for protein expression, metabolic engineering, therapeutic development, education, and more. The advantages of CFPS over in vivo protein expression include its open system, the elimination of reliance on living cells, and the ability to focus all system energy on production of the protein of interest. Over the last 60 years, the CFPS platform has grown and diversified greatly, and it continues to evolve today. Both new applications and new types of extracts based on a variety of organisms are current areas of development. However, new users interested in CFPS may find it challenging to implement a cell-free platform in their laboratory due to the technical and functional considerations involved in choosing and executing a platform that best suits their needs. Here we hope to reduce this barrier to implementing CFPS by clarifying the similarities and differences amongst cell-free platforms, highlighting the various applications that have been accomplished in each of them, and detailing the main methodological and instrumental requirement for their preparation. Additionally, this review will help to contextualize the landscape of work that has been done using CFPS and showcase the diversity of applications that it enables.

## 1. Introduction

Cell-free protein synthesis (CFPS) emerged about 60 years ago as a platform used by Nirenberg and Matthaei to decipher the genetic code and discover the link between mRNA and protein synthesis [1]. Since this discovery, the CFPS platform has grown to enable a variety of applications, from functional genomics to large-scale antibody production [2,3]. Currently, CFPS has been implemented using cell extracts from numerous different organisms, with their unique biochemistries enabling a broad set of applications. In an effort to assist the user in selecting the CFPS platform that is best suited to their experimental goals, this review provides an in-depth analysis of high adoption CFPS platforms in the scientific community, the applications that they enable, and methods to implement them. We also review applications enabled by low adoption platforms, including applications proposed in emerging platforms. We hope that this will simplify new users’ choice between platforms, thereby reducing the barrier to implementation and improving broader accessibility of the CFPS platform.

**Figure 1:**
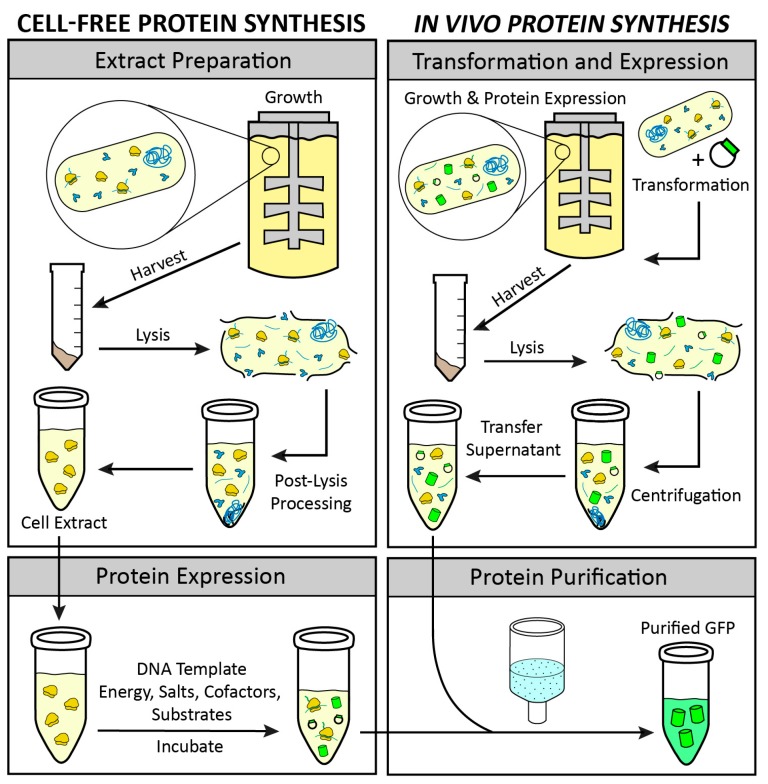
A comparison of cell-free and in vivo protein synthesis methods. Through visualization of the main steps of in vitro and in vivo protein expression, the advantages of cell-free protein synthesis emerge. These include the elimination of the transformation step, an open reaction for direct manipulation of the environment of protein production, the lack of constraints based on the cell’s life objectives, the channeling of all energy toward production of the protein of interest, and the ability to store extracts for on-demand protein expression. Green cylinders represent synthesized green fluorescent protein (GFP).

**Figure 2:**
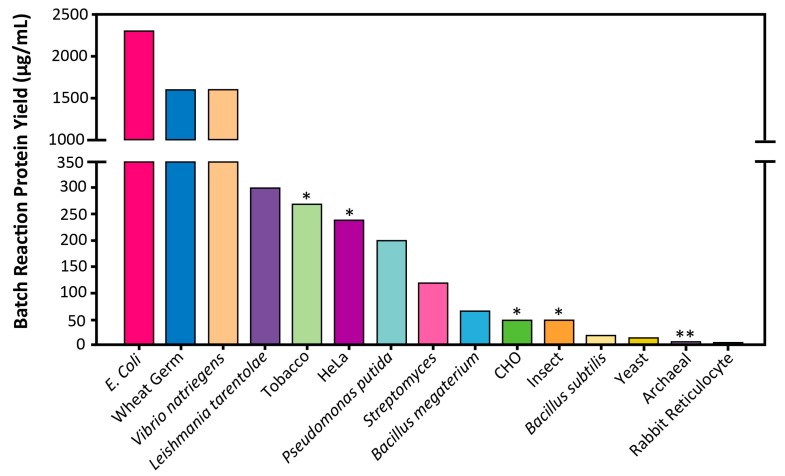
Comparison of protein yields across cell-free platforms. The volumetric yield of each platform is reported for batch reactions producing GFP. Platforms that report volumetric yield for reporter proteins luciferase (*) or ChiAΔ4 (**) are indicated. Information for batch mode protein yields of the *Arabidopsis* and *Neurospora crassa* platforms was not found. Yields were obtained from the following sources: *E. coli* [8]*,* wheat germ [9], *Vibrio natriegens* [10], *Leishmania tarentolae* [11], tobacco [12], HeLa [13], *Pseudomonas putida* [14], *Streptomyces* [15], *Bacillus megaterium* [16], Chinese hamster ovary [17], insect [18], *Bacillus subtilis* [16], yeast [19], archaeal [20], and rabbit reticulocyte [21].

**Figure 3:**
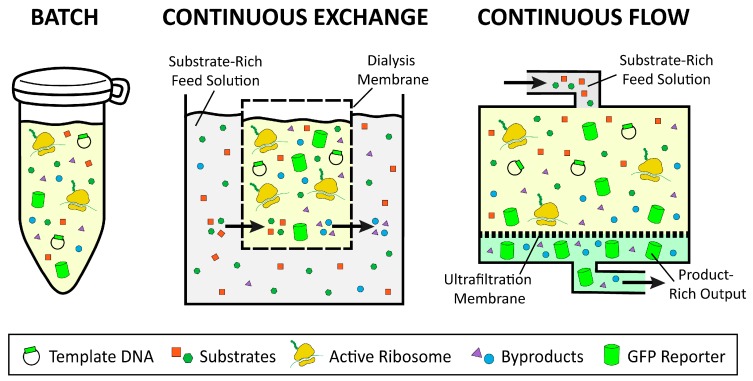
Comparison of batch, continuous flow, and continuous exchange reaction formats. Batch reactions contain all the necessary reactants within a single reaction vessel. Continuous exchange formats utilize a dialysis membrane that allows reactants to move into the reaction and byproducts to move out, while the protein of interest remains in the reaction compartment. Continuous flow formats allow a feed solution to be continuously pumped into the reaction chamber while the protein of interest and other reaction byproducts are filtered out of the reaction.

**Figure 4:**
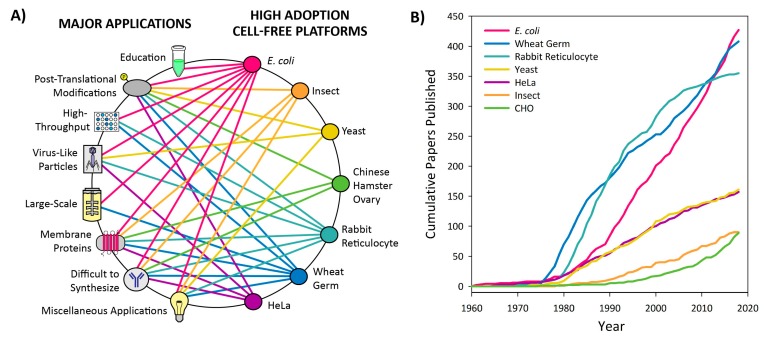
High adoption cell-free platforms and their applications. (**A**) Web of the applications enabled by high adoption cell-free platforms. The connections shown are based on applications that have been published for each respective platform. Applications under “difficult to synthesize proteins” include the production of antibodies, large proteins, ice structuring proteins, and metalloproteins. Miscellaneous applications include studies of translational machinery, genetic circuits, metabolic engineering, and genetic code expansion. (**B**) Cumulative number of peer-reviewed publications over the last 60 years for high adoption platforms. The metric of cumulative publications by platform is used to indicate which platforms are most utilized, with platforms having over 25 papers categorized as high adoption. These data were generated by totaling papers from a PubMed Boolean search of the following: (“cell free protein synthesis” OR “in vitro transcription translation” OR “in vitro protein synthesis” OR “cell free protein expression” OR “tx tl” OR “cell-free translation”) AND “platform name.” The platform name used for each search corresponds to the name listed in the graph’s key. This information was collected on 23 December 2018, and the search results for each platform can be found in Supplemental Information. While this metric is an indicator of the level of adoption for each platform, it does suffer from false positive search results, such as papers reporting studies in which the researchers produce recombinant proteins from the organism of interest rather than from cell extract derived from that organism.

**Figure 5:**
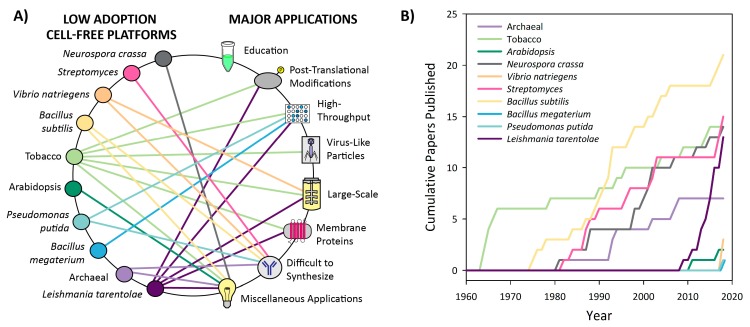
Low adoption cell-free platforms and their applications. (**A**) Web of the applications enabled by low adoption cell-free platforms. Connections shown are based on applications that have been published or that have been proposed in publications. Applications under “difficult to synthesize proteins” include high GC content proteins, antimicrobial peptides, pharmaceutical proteins, and thermophilic proteins. Miscellaneous applications include studies of translational machinery, investigation of antibiotic resistance, genetic circuits, metabolic engineering, and genetic code expansion. (**B**) Cumulative number of peer-reviewed publications over the last 60 years for low adoption platforms. We have used the metric of cumulative publications to indicate which platforms are less utilized and have categorized platforms with under 25 papers as low adoption platforms. These data were generated by totaling papers from a PubMed Boolean search of the following: (“cell free protein synthesis” OR “in vitro transcription translation” OR “in vitro protein synthesis” OR “cell free protein expression” OR “tx tl” OR “cell-free translation”) AND “platform name.” The platform name used for each search corresponds to the name listed in the graph’s key. While this metric is an indicator of the level of adoption for each platform, it does suffer from inconsistencies due to irrelevant search results, such as papers reporting studies in which the researchers produce proteins from the organism of interest rather than from cell extract derived from the organism. This inconsistency was significant for platforms with fewer papers, so we pursued data curation to remove irrelevant papers and add in missing papers. This information was collected on 23 December 2018, and curated search results for each platform can be found in Supplemental Information, where red indicates that the paper was removed from the search results and green indicates that the paper was added to the search results.

The growing interest in CFPS is the result of the key advantages associated with the open nature of the platform. The CFPS reaction lacks a cellular membrane and a functional genome, and consequently is not constrained by the cell’s life objectives [4]. Therefore, the metabolic and cytotoxic burdens placed on the cell when attempting to produce large quantities of recombinant proteins in vivo are obviated in CFPS [5]. The CFPS platform is amenable to direct manipulation of the environment of protein production because it is an open system (Figure 1). In some cases higher protein titers can be achieved using CFPS because all energy in the system is channeled toward producing the protein of interest (Figure 2) [6]. Moreover, CFPS reactions are flexible in their setup, allowing users to utilize a variety of reaction formats, such as batch, continuous flow, and continuous exchange, in order to achieve the desired protein titer (Figure 3). These advantages make CFPS optimally suited for applications such as the production of difficult-to-synthesize proteins, large proteins, proteins encoded by high GC content genes, membrane proteins, and virus-like particles (Figure 4A and Figure 5A). The scalable nature of CFPS allows it to support the discovery phase through high-throughput screening as well as the production phase through large-scale biomanufacturing. Additional high impact applications include education, metabolic engineering, and genetic code expansion. 

**Figure 6:**
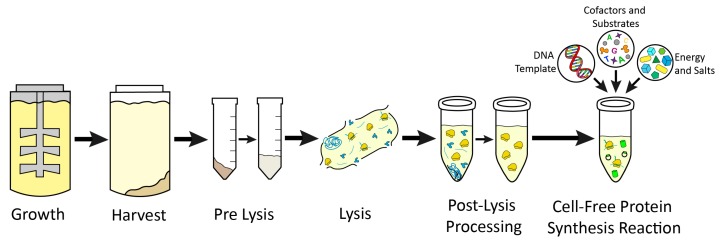
General workflow for preparation of cell-free extract and set up of CFPS reactions. A visualization from cell growth to the CFPS reaction is depicted above for a new user, highlighting the main steps involved.

While the number of cell-free platforms based on different organisms has grown substantially since its conception, the basic steps for successful implementation of a cell-free platform are analogous across platforms (Figure 6). In brief, users must culture the cell line of interest from which transcription and translation machinery are to be extracted. Next, the user must lyse the cells while maintaining ribosomal activity in the lysate, prepare cell extract by clarifying the lysate through various methods, and then utilize the prepared cell extract in CFPS reactions to synthesize the protein of interest. These basic steps have many nuanced variations from platform to platform, and even within platforms. Lysis methods in particular are extremely variable and commonly used methods include homogenization, sonication, French press, freeze thaw, nitrogen cavitation, bead beating [7]. Extract preparation varies by centrifugation speeds, run off reactions, dialysis, or treatment with nucleases to remove endogenous DNA or RNA. Here, we report methodologies used most commonly for obtaining highest volumetric yields of the target protein (Table 1, Table 2 and Table 3). We also report low adoption platforms including emerging platforms that adapt these methods for continued innovations in CFPS.

Based on nearly 60 years of literature, we have divided CFPS platforms into two categories: high adoption and low adoption platforms. The latter also includes emerging platforms. High adoption platforms for CFPS are based on extracts from the following cell lines: *Escherichia coli*, *Spodoptera frugiperda* (insect), *Saccharomyces cerevisiae* (yeast), Chinese hamster ovary, rabbit reticulocyte lysate, wheat germ, and HeLa cells. These platforms have been well optimized and utilized since their conception and are most easily implemented by new users due to the breadth of supporting literature (Figure 4). Platforms that have experienced low adoption to date include *Neurospora crassa, Streptomyces*, *Vibrio natriegens*, *Bacillus subtilis*, Tobacco, *Arabidopsis*, *Pseudomonas Putida*, *Bacillus megaterium*, Archaea, and *Leishmania tarentolae*. These platforms have not been widely used or developed, and some have even emerged in the last two years as promising candidates for new applications (Figure 5). Trends in CFPS literature demonstrate that there is continued development and optimization of platforms, and the emerging platforms are likely to be the source of rapid innovations. We also anticipate significant development toward the broad dissemination and utilization of CFPS platforms.

## 2. CFPS Reaction Formats

As an open and highly personalized platform, CFPS reactions can be executed in a variety of formats, including coupled, uncoupled, batch, continuous flow, continuous exchange, lyophilized, or microfluidic formats depending on the needs of the user. Additionally, there are a variety of commercial CFPS kits available for users looking to implement CFPS quickly, without the need for long-term or large-scale usage. Here we describe the differences and utility of each format. 

### 2.1. Commercial Systems

Many of the high adoption CFPS platforms have been commercialized as kits available for users to quickly leverage the advantages of CFPS for their research. This has generally been the best option for labs lacking the access and technical expertise necessary to produce their own cell extracts. Commercial kits enable users to implement CFPS easily, but for extensive usage, they may not be cost-effective. For example, in house prepared *E. coli* CFPS costs about $0.019/μL of reaction while commercial lysate-based kits cost $0.15–0.57/μL of reaction [22]. Currently commercial kits exist for *E. coli* (New England Biolabs, Promega, Bioneer, Qiagen, Arbor Biosciences, ThermoFisher, Creative Biolabs), rabbit reticulocyte (Promega, Creative Biolabs), wheat germ (Promega, Creative Biolabs), *Leishmania tarentolae* (Jena Bioscience)*,* insect (Qiagen, Creative Biolabs), Chinese hamster ovary (Creative Biolabs), HeLa (ThermoFisher, Creative Biolabs), and plant cells (LenioBio).

In addition to cell-extract-based CFPS kits, the PURExpress kit is comprised of a reconstitution of purified components of the transcription and translation machinery from *E. coli*. Specifically, the PURE (**p**rotein synthesis **u**sing **r**ecombinant **e**lements) system utilizes individually purified components in place of cell extract. These include 10 translation factors: T7 RNA polymerase, 20 aminoacyl-tRNA synthetases, ribosomes, pyrophosphatase, creatine kinase, myokinase, and nucleoside diphosphate kinase [23,24]. This system requires overexpression and purification of each component but benefits from the absence of proteases and nucleases, and the defined nature of the system. Overall, the PURE system allows for high purity and somewhat easier manipulation of the reaction conditions than even cell-extract-based CFPS [23]. Moreover, if all synthesized components are affinity-tagged, they can be easily removed post-translationally to leave behind the protein of interest [24]. This system may provide advantages for the synthesis of properly folded proteins with supplemented chaperones, genetic code expansion, and display technologies [23,24,25]. The PURE system would be significantly more time-consuming to produce in-house but is available commercially (New England BioLabs, Creative Biolabs, Wako Pure Chemical Industries). However, these kits are expensive ($0.99/μL of reaction) when compared to both in-house and commercially available extract-based CFPS [22]. They are also significantly less productive (~100 µg/mL) than their extract-based *E. coli* CFPS counterpart (Figure 2) [23,26].

### 2.2. Coupled and Uncoupled Formats

CFPS reactions can be performed in coupled or uncoupled formats, and the choice is dependent on the platform being used and the user’s needs. Coupled reactions allow transcription and translation to take place within a single tube, such that the supplied DNA template can be transcribed into mRNA, which is then translated into protein within a one-pot reaction. The advantage of coupled CFPS is the ease of reaction setup, but it may result in suboptimal yields for eukaryotic platforms. Uncoupled reactions typically consist of an in vitro transcription reaction followed by mRNA purification; the purified transcripts are then supplied to the cell-free translation reaction containing the cell extract for production of the protein of interest. Uncoupled reactions are more often utilized in eukaryotic CFPS platforms due to mRNA processing for more efficient translation of certain transcripts. As an example, pseudouridine modification for mRNA in the rabbit reticulocyte platform has been demonstrated to enhance translation [27]. Uncoupled reactions also allow for different conditions between transcription and translation reactions, which can improve yields [9]. Uncoupled reactions can be achieved in any platform by supplying the reaction with mRNA instead of DNA, but mRNA can be more difficult to handle and does degrade more quickly in the CPFS reaction [28].

### 2.3. Batch, Continuous Flow, and Continuous Exchange Formats

CFPS reactions can be performed in batch format for simplified setup, or in continuous formats for improved protein yields. Reactions are most easily, quickly, and cheaply set up in batch format because all necessary reactants are added to a single tube and incubated for protein synthesis to occur (Figure 3). However, the duration of a batch reaction is dependent on the amount of substrate available and the amount of inhibitory byproduct produced, which can result in low yields for some platforms (Figure 2). On the other hand, continuous flow and continuous exchange CFPS reactions utilize a two-chamber system to supply reactants and remove products, for increased reaction duration and higher protein yields [29,30,31,32]. In continuous exchange cell-free (CECF), the CFPS reaction is separated from a reactant-rich feed solution via a semi-permeable membrane, such that new reactants move into the reaction and byproducts move out, while the protein product remains in the reaction compartment (Figure 3) [31]. For continuous flow cell-free (CFCF), the feed solution is continuously pumped into the reaction chamber, while the protein of interest and other byproducts are pushed out through an ultrafiltration membrane (Figure 3) [33]. 

Batch reactions are well suited to platforms that exhibit high protein yields and to applications that require simple and fast setup (Figure 2). These applications may include high-throughput screening and education. Moreover, batch reactions can be easily scaled up in platforms such as *E. coli* and wheat germ, due to the ability to scale growth and reaction setup linearly. Platforms such as Chinese hamster ovary, yeast, and rabbit reticulocyte, which suffer from low protein yields, may require a CFCF or CECF setup to generate sufficient amounts of protein. Continuous formats have already been successfully constructed in Chinese hamster ovary, insect, *E. coli*, wheat germ, and yeast [30,32,34,35,36,37]. For example, continuous formats have allowed for the synthesis of 285 μg/mL of human EGFR to be produced by the insect platform, 980 μg/mL of membrane protein in the Chinese hamster ovary platform, and up to 20,000 μg/mL of protein in wheat germ [9,38,39]. Continuous formats may also be used for large-scale protein synthesis reactions in industrial applications [38,39]. Scale-up of CFPS reactions will be discussed in more detail in Section 3.2.5 titled “Large-Scale.”

### 2.4. Lyophilization

Lyophilization, or freeze-drying, has been used as a technique to stabilize cell extracts for long-term and higher temperature storage, and to provide a condensed format to reduce necessary storage space. By overcoming the cold chain, lyophilization could help enable applications such as on-demand biosensors for diagnostics, therapeutic production in remote locations, personalized medicines, and more [40]. Lyophilization has only been heavily pursued for *E. coli* extract thus far, with some additional work done on the lyophilization of other CFPS reagents and the addition of lyoprotectant additives, and with preliminary work done in wheat germ [41].

Traditionally, aqueous cell-extract is stored at −80 °C, and its activity is reduced by 50% after just one week of storage at room temperature, with all activity lost after a month [42]. In comparison, lyophilized extract maintains approximately 20% activity through 90 days of storage at room temperature. Importantly, the process of lyophilization does not negatively impact reaction yields. A CFPS reaction run directly after lyophilization could achieve the same yields as an aqueous reaction [42]. Lyophilized extract also reduces storage volume to half and mass to about one-tenth [42]. Importantly, the process of lyophilization itself does not negatively impact extract productivity [43]. Lyophilization of extract has also been done on paper, rather than in a tube, to further improve storage and distribution of cell-free technology [44,45].

Some work has been done to test the viability of lyophilizing CFPS reagents necessary for a phosphoenolpyruvate-based reaction setup. These reagents were lyophilized with or without the extract, and while viability was improved over aqueous storage of the reagents at higher temperatures, the combined extract and reagent mixture posed new challenges to the handling of the lyophilized powder due to the resulting texture [42]. Other users have lyophilized the template of interest separately from otherwise fully prepared CFPS reaction for classroom applications, such that the template is simply rehydrated and added to the reaction pellet to begin protein synthesis [46,47]. Additionally, lyoprotectants for cell-free applications have been briefly screened, including sucrose, which provided no obvious benefits to storage stability [42].

### 2.5. Microfluidics Format

The growing field of microfluidics consists of many broad methodologies that generally involve the manipulation of fluids on the micron scale on devices with critical dimensions smaller than one millimeter [48]. These devices, when paired with cell-free extracts, provide cost-effective and rapid technologies capable of high-throughput assays to generate protein in an automated series of channels that often consist of mixers, reactors, detectors, valves, and pumps on a miniaturized scale [49]. The utilization of microfluidics to pioneer biomedical and diagnostic approaches for sensing and monitoring environmental and health issues has been achieved within *E. coli*, wheat germ, and insect platforms [49]. Examples of applications that utilize the microfluidics format include both the *E. coli* and wheat germ platforms to test for the presence of ricin in orange juice and diet soda through the generation of a reporter protein [50,51]. The insect platform was also used in a Transcription-RNA Immobilization and Transfer-Translation (TRITT) system for the production of a cytotoxic protein with simultaneous non-standard amino acid incorporation for fluorescence labeling [52].

## 3. Applications of Cell-Free Protein Synthesis

### 3.1. Introduction to Platform Categorization

In the 60 years since cell-free protein synthesis emerged, a multitude of platforms have been developed based on cell extracts from a variety of organisms. These include extracts from bacterial, archaeal, plant, mammalian, and human cell lines. Each resulting platform varies in ease of preparation, protein yields, and in possible applications resulting from the unique biochemistry of the given organism. In this review, we have divided these various platforms into two categories: high adoption and low adoption (Figure 4 and Figure 5 and Supplementry Materials). The platforms have been categorized based on our understanding of their development and the degree to which they have been adopted by the field, as quantified by the number of peer-reviewed publications that utilize each platform (Figure 4B and Figure 5B). This categorization allows new users to identify platforms that have been best established and to explore the applications that they enable. We believe that the depth of literature available for these platforms makes them optimally suited for newer users. Low adoption platforms may be particularly useful for niche applications, but have not been optimized thoroughly, or are currently emerging in the field. Therefore, these platforms may be more difficult to implement due to minimal development. Platforms with fewer than 25 peer-reviewed publications to date have been categorized as “low adoption.”

### 3.2. High Adoption Platforms

High adoption platforms include those based on *E. coli*, insect, yeast, Chinese hamster ovary, rabbit reticulocyte lysate, wheat germ, and HeLa cells (Figure 4). These platforms have been utilized for a variety of applications and have withstood the test of time to establish their utility and versatility within the CFPS field. Briefly, bacterial CFPS platforms including *E. coli* tend to have higher protein yields and are typically easier and faster to prepare (Figure 2). However, they can be limited in some applications such as post-translational modifications, membrane protein synthesis, and other difficult-to-synthesize proteins. In such cases, eukaryotic platforms are well suited for the synthesis of traditionally difficult proteins without requiring significant augmentation or modifications to the cell extracts. Within the eukaryotic platforms, wheat germ provides the highest productivity; rabbit reticulocyte, Chinese hamster ovary, HeLa, yeast, and insect platforms give significantly lower yields but may have other advantages for post-translation modifications, membrane proteins, or virus-like particles. In order to enable users to select a platform that will support their experimental goals, the discussion of high adoption platforms is application-driven. For each application, the relevant platform and reaction formats are discussed.

#### 3.2.1. Education

The open nature of the CFPS system and the resulting access to directly manipulate cellular machinery enables inquiry-based learning opportunities that make CFPS particularly suitable for the classroom. The first application of CFPS technology for education is the BioBits kits, which were tested with students of various ages [46,53]. These kits offer versatile experimental options and are relatively inexpensive (about $100 for a class set). The BioBits Bright and Explorer kits represent the diversity of classroom experiments and applications that can be enabled by CFPS, from production of fluorescent proteins, to hydrogel production, and to identification of fruit DNA [46,53]. These possibilities show that CFPS enables inquiry-based learning of concepts in biochemistry in a hands-on fashion. The stability of CFPS classroom kits is achieved through lyophilization of reaction components. More information on lyophilization of CFPS can be found in Section 2.4, titled “Lyophilization.”

#### 3.2.2. Post-Translational Modifications (PTMs)

Post-translational modifications (PTMs) can greatly affect protein folding, activity, and stability, which may be essential for therapeutic proteins, membrane proteins, and virus-like particles, among others [54]. As such, the ability to incorporate various post-translational modifications (PTMs) into the protein of interest is a key consideration when choosing a CFPS platform. PTMs achieved through genetic code expansion will be discussed in Section 3.2.8.4 “Genetic Code Expansion.” Here, we cover some key PTMs possible in each high adoption platform and the necessary modifications of the platform that may be needed to achieve them. A key consideration is that platforms with endogenous microsomes demonstrate a greater capacity to support PTMs. This makes platforms such as Chinese hamster ovary, HeLa, and insect well-suited for this application, as endogenous microsomes are formed from endoplasmic reticulum and maintained during extract preparation. However, when endogenous microsomes are utilized, a new “black box” is introduced to the system, which limits user control and restricts PTM choice to those innately possible in the cell line [54].

In the rabbit reticulocyte platform, a variety of PTMs have been investigated, including glycosylation, cleavage of signal proteins, prenylation, and disulfide bond formation [55,56,57,58,59,60,61]. However, rabbit reticulocyte extract requires the addition of exogenous microsomes for PTM incorporation. The platform has also been used to probe the specificity of signal sequence differences between glycosylphosphatidylinositol anchoring and translocation to the ER lumen, which was found to be sensitive to even single residue changes [56].

Insect cell CFPS, which contains endogenous microsomes, allows for signal peptide cleavage, glycosylation, phosphorylation, *N*-myristoylation, *N*-acetylation, prenylation, and ubiquitination [62,63,64,65,66,67,68,69,70]. These possible PTMs are similar to those of rabbit reticulocyte and other mammalian platforms [62]. Disulfide bond formation can also be achieved in these platforms by preparing the cell extract under non-reducing conditions, and adding glutathione along with protein disulfide isomerase to the reactions [71]. The insect cell-free platform was even used to discover new proteins containing a PTM of interest. These techniques utilized MALDI-TOF MS screening of a library of metabolically labeled cDNA clones with motifs matching *N*-myristoylated proteins to determine which were most susceptible to this PTM [72].

Some PTMs can be achieved in *E. coli*-based CFPS, but this application is generally more technically challenging due to a lack of endogenous microsomes and the limited number of PTMs possible in bacteria when compared to eukaryotes [64]. However, utilization of *E. coli* remains advantageous in terms of overall protein yields and ease of extract preparation, which have prompted the development of PTMs in this platform. The open nature of the reaction enables users to tune redox conditions to make disulfide bond formation feasible in this platform. Additionally some N-linked glycosylation has been made possible through the supplementation of glycosylation machinery [73]. Glycosylation was first achieved through the addition of purified glycosylation components after completed cell-free translation, which was effective, but relatively time-consuming [74]. More recently, oligosaccharyltransferases have been synthesized in CFPS and shown to be active in in vitro glycosylation without the need for purification [75]. Furthermore, *E. coli* strains that have been optimized for glycoprotein synthesis have been used to prepare cell-free extract, such that glycosylation can be pursued in a one-pot system [54].

Chinese hamster ovary extract contains endogenous microsomes, which provide glycosyltransferases for glycoprotein synthesis, chaperones, and other molecules necessary for disulfide bridge formation [34]. Yeast has also been a platform of interest for protein glycosylation, with glycosylation achieved when a completely homologous system was used and yeast microsomes were added. However, yields in this platform are much lower than in *E. coli* [76]. The wheat germ platform also requires exogenous microsome addition, which has allowed for some PTMs to be incorporated [64,77]. A human-based hybridoma-cell extract platform, similar to that of HeLa cell-based extracts, was able to glycosylate human immunodeficiency virus type-1 envelope protein 120 [78].

#### 3.2.3. High-Throughput Screening

The ability to achieve high-throughput protein production is a major advantage of CFPS, as it enables rapid production and screening of a variety of protein products much faster than in in vivo protein expression (Figure 1). Coupled CFPS allows for DNA templates to be plugged in directly without the need for cell transformation/transfection, and in some cases, assays of protein products can be done without the need for purification, creating a powerful one-pot system [28]. A key application of high-throughput CFPS is functional genomics, which allows for the elucidation of new genes and their corresponding protein function. High-throughput screening can be pursued in any platform, but most often utilizes *E. coli*, wheat germ, and rabbit reticulocyte extracts. Here we will discuss some specific examples of CFPS for high-throughput applications.

The *E. coli* platform has been widely used and is well-developed, with relatively simple extract preparation and high yields making it a prime candidate for high-throughput synthesis (Table 2, Figure 2). One notable application of *E. coli-*based CFPS is the ability to screen antibody mutant libraries in rapid design–build–test cycles for antibody engineering. The best mutants could later be scaled up in the same platform for industrial level synthesis (see Section 3.2.5, titled “Large-Scale”) [2]. Additionally, the *E. coli* platform has been used for high-throughput functional genomics to identify numerous gene products involved in complex metabolic systems that result in protein accumulation and folding in vitro [3].

While high-throughput applications commonly utilize *E. coli*, the eukaryotic wheat germ platform has advantages for synthesis of soluble, active protein, making it better suited for structural and functional analysis of certain proteins in CFPS [79]. The wheat germ platform has shown the capacity to perform as a “human protein factory” when it was utilized in an attempt to produce 13,364 human proteins. Using the versatile Gateway vector system to generate entry clones allowed for successful synthesis of 12,996 of the human proteins, with many displaying successful function [80].

CFPS from rabbit reticulocyte extract can also be used in a high-throughput fashion for protein microarrays, in order to study protein function, interaction, and binding specificity [81,82]. Ribosome and mRNA display technologies as well as in vitro compartmentalization are also possible in the rabbit reticulocyte platform and allow for genes to be linked to their protein products for functional genomic studies [83,84]. Lastly, the Chinese hamster ovary platform is a candidate for high-throughput synthesis, but examples of implementation have not been demonstrated to date [34,85].

#### 3.2.4. Virus-Like Particles

Virus-like particles (VLPs) are capsids of viruses lacking genomic material, meaning that they are a highly organized and symmetrical aggregations of proteins, capable of carrying molecules of interest within them. As such, production of VLPs allows for study of viral assembly, the creation of effective vaccines, drug delivery using encapsulation, and materials science applications [86]. While VLPs can be produced in vivo, production in CFPS platforms offers advantages including the ability to synthesize toxic VLPs and to manipulate the redox conditions of the reaction for proper disulfide bond formation, which may be essential for thermal stability [86]. The versatility of the CFPS reaction also allows for a single, more robust platform capable of producing many types of VLPs at scalable, higher yields and with easier modification of reaction setup than would be possible in vivo [87].

A variety of CFPS platforms have been used to produce many different VLPS, including *E. coli*, HeLa, rabbit reticulocyte, and yeast. The *E. coli* platform has been used to optimize disulfide bond formation in Qβ VLPs by expression without change to the redox state of the reaction and subsequent exposure to diamide to form disulfide bonds post-assembly, as VLP formation would occur naturally. The Qβ VLP has also been co-expressed with A2 protein, which naturally occurs in the full virus for infection and competitive inhibition purposes [88]. Additionally, human hepatitis B core antigen was produced by supplementation with disulfide forming agents glutathione and disulfide isomerase [86]. MS2 bacteriophage coat proteins have also been expressed in high yields using *E. coli-*based CFPS [87]. Both MS2 and Qβ VLPs have been produced with non-standard amino acid enabled click chemistry, allowing proteins, nucleic acids, and polymer chains to be attached to the surface of the VLPs [89]. In the last year, the *E. coli* platform has enabled the production of the largest biological entities thus far in a CFPS platform: fully functional T7 and T4 bacteriophages [90].

The HeLa cell-based CFPS platform has been used for poliovirus synthesis [91]. Rabbit reticulocyte CFPS has enabled viral assembly studies of HIV Gag protein assembly, which forms immature but fully spherical capsids in CFPS [92]. Furthermore, adenovirus type 2 fibers are able to self-assemble into trimers in rabbit reticulocyte CFPS reactions and hepatitis C core proteins are able to form into capsids, which is not seen in mammalian cell cultures [93,94]. The yeast platform has allowed for optimization of translation of VLPs such as human papillomavirus 58 (HPV 58). Synthesis of this VLP through CFPS could enable the study of capsid assembly and encapsulation mechanisms for HPV [95,96].

#### 3.2.5. Large-Scale

The demonstrations of implementing CFPS from a high-throughput scale for discovery to a manufacturing scale have expanded the utility of this platform [2,97]. Users interested in leveraging this capacity for applications such as the production of antibodies and industrial enzymes, as well as CFPS kit production for field or educational uses, should consider the technical details of scaling up the entire workflow for CFPS (Figure 6). This begins with the capacity to scale cell growth, as well as scaling extract preparation. Platforms that enable this scalability include *E. coli*, wheat germ, and rabbit reticulocyte (Table 1 and Table 2) [97,98,99]. The insect, yeast, and Chinese hamster ovary platforms may also be amenable to scale-up in culture growth, as they are fermentable, but large-scale extract preparation has not been well studied to date [100].

Next, platforms must have scalable CFPS reactions that maintain volumetric protein yields even in large-scale reactions. *E. coli* CFPS has been shown to scale over many orders of magnitude in batch format, from reactions as small as 10 µL to as large as 100 L [97]. Within this range of reaction sizes, volumetric protein yields remain constant if the proper reaction vessel is used. For example, reactions up to 100 µL can be run in 1.5–2 mL microcentrifuge tubes, while reaction over 100 µL should be run in 24-well microtiter plates or a similar thin-layer format [31,101,102]. For liter-scale reactions, bioreactors and fermenters have been used [2,97]. The importance of vessel size for scale-up of batch reactions is due in part to the need for proper oxygen exchange, such that increasing the surface area to volume ratio of the reaction can significantly improve reaction yields [31,102]. The scalability of the *E. coli* platform and discovery of cost-effective metabolisms makes it well suited for industrial applications, as has been demonstrated by companies such as Sutro Biopharma, who use CFPS to produce large batches of antibodies in vitro [103,104].

The wheat germ platform has been used for reaction scale-up through a robotic discontinuous batch reaction that can perform reactions up to 10 mL in volume. This setup is capable of producing at least 2 mg/mL of the protein of interest, including DCN1, involved in ubiquitination, human sigma-1 receptor, and bacteriorhodopsin transmembrane proteins. This system utilizes multiple cycles of concentration, feed buffer addition, mRNA template addition, and incubation to achieve high protein yields with minimized extract usage, an idea similar to continuous flow cell-free (CFCF) and continuous exchange cell-free (CECF) [30,99]. CECF and CFCF formats may also be used to scale up reaction size and increase protein yields as discussed in Section 2.3 “Batch, Continuous Flow, and Continuous Exchange Formats.” Continuous formats have been pursued in Chinese hamster ovary, insect, *E. coli*, wheat germ, and yeast [30,32,34,35,36,37]. Overall, the *E. coli* and wheat germ platforms are most amenable to large-scale synthesis, as scale-up of the entire CFPS workflow has been demonstrated.

#### 3.2.6. Membrane Proteins

The study of membrane proteins is an integral component of proteomics due to their high abundance within organisms. Approximately 25% of all sequenced genes code for hydrophobic proteins that integrate themselves into cell membranes [105]. Membrane proteins serve a plethora of functions within cells including cell recognition, immune response, signal transduction, and molecule transport. However, expressing these complete proteins in vivo in their correct conformation often poses a challenge due to the naturally low abundance during expression, high hydrophobicity, the necessity of translocation into the membrane, and the impact to the host cell’s membrane integrity.

CFPS platforms are able to circumvent these challenges by avoiding dependence on the structural integrity of the cell membrane via the non-membrane bound system [106]. In addition, the supplementation of microsomes, vesicle-like structures, or the presence of endoplasmic reticulum fragments during extract preparation (endogenous microsomes) allows membrane proteins to correctly fold and incorporate themselves into these structures during protein synthesis. Namely, the HeLa, Chinese hamster ovary, and insect platforms all contain endogenous microsomes formed via rupturing of the endoplasmic reticulum during extract preparation. These platforms have successfully expressed a number of membrane proteins ranging from a two-transmembrane malarial protein (HeLa), to epidermal growth factor receptor proteins (Chinese hamster ovary), and finally to a KcsA potassium channel (insect) [39,107,108].

Platforms that require exogenous addition of microsomal structures for membrane protein expression include rabbit reticulocyte, wheat germ, and *E. coli*. The rabbit reticulocyte platform, with the supplementation of semipermeable cells, has been demonstrated to properly express MHC class I heavy chain membrane proteins in their correct conformations [109]. The wheat germ platform has successfully expressed human, mouse, and mycobacterium desaturase complexes with the addition of liposomes, as well as plant solute transporters, using a similar strategy [110,111]. The *E. coli* platform has shown expression of a wide variety of membrane proteins including pores, channels, transporters, receptors, enzymes, and others while utilizing the exogenous addition of synthetic liposomes [106,112]

#### 3.2.7. Difficult to Synthesize Proteins

The advantages of cell-free protein synthesis over in vivo protein synthesis, such as the open reaction and absence of living cells, allow for the production of proteins that would be difficult to manufacture in vivo due to the burden on the cell and inability to manipulate the environment of protein production (Figure 1). Such examples include antibodies, large proteins, ice structuring proteins, and metalloproteins.

Other applications, such as expression of proteins from high GC content templates (Section 3.3.2, titled “Streptomyces*”* and Section 3.3.7. titled *“Pseudomonas putida”*) and thermostable proteins (Section 3.3.9, titled “Archaeal”), will be discussed in the low adoption section.

##### 3.2.7.1. Antibodies

The production of functional antibodies and antibody fragments in vitro using CFPS has the potential to allow for simplification of the antibody production process for more rapid manufacturing. This advantage is due in part to the open system, which can easily be modified from case to case for the production of active antibodies using rapid design–build–test cycles and modification of the redox potential of the reaction. Antibody production has taken place in rabbit reticulocyte, *E. coli*, Chinese hamster ovary, wheat germ, and insect platforms [100,113].

One of the first instances of antibody production in a CFPS platform was the synthesis of the light chain of mouse Ig in rabbit reticulocyte [114]. Later on, the rabbit reticulocyte platform was also used to synthesize the scFv-toxin fusion protein, which contains both single-chain and gamma globulin antibodies [115,116].

Previous studies in *E. coli* have shown that protein disulfide isomerase for disulfide bond shuffling is important for active antibody formation, while addition of DsbA, a thiol disulfide oxidoreductase, does not improve active yield. This study also found that the addition of chaperones helped to increase soluble yields but not functional yield [117]. Moreover, cell-free expression has been used to overcome low yields that occur in vivo with rearrangement of variable regions [118]. In *E. coli*, synthesis of full-length correctly folded and assembled antibodies has been accomplished in a range of scales. Fab antibodies have been produced with 250 µg/mL yields in reaction scales from 60 µL to 4 L, and scFv antibodies with yields up to 800 µg/mL in reaction scales from 10 µL to 5 L. CFPS reactions containing iodoacetamide, protein disulfide isomerase, and both oxidized and reduced glutathione are used to increase active yields. These yields were also improved for industrial production by codon optimization, translation initiation optimization, and temporal assembly optimization. This demonstrates the power of CFPS for antibody production in industry as well as in screening and optimization [2]. The *E. coli* platform has also allowed for the synthesis of IgG antibody drug conjugates using genetic code expansion and iodoacetamide-treated extract supplemented with glutathione [119]. Other antibodies including the Fab fragment of 6D9, scFv to Erb-2, and even gram per liter IgG yields have been obtained in *E. coli* [120,121,122].

The Chinese hamster ovary platform has recently emerged as an easily optimizable platform for high yield synthesis of monoclonal antibodies (mAbs). Using a commercially available extract, successful synthesis of aglycosylated, active mAbs in yields greater than 100 μg/mL has been accomplished. The process has been taken a step further by exploring the utility of the platform for ranking yields of candidate antibodies [103]. Antibody production has also been achieved in wheat germ by lowering the concentration of DTT in the reaction or by adding protein disulfide isomerase and oxidized and reduced glutathione [123].

In the insect platform, which contains its own microsomes, adjustment of the redox potential in the reaction by omitting DTT and including glutathione allowed for the creation of antibody-enriched vesicles containing functional antibodies. This technique is notable as it mimics synthesis of antibodies as it would occur in living cells and allows for the vesicles and antibodies to be easily and efficiently separated from the CFPS reaction [124]. Moreover, single-chain antibody fragments with non-standard amino acid incorporation have been produced in the insect platform via translocation to microsomes [125]. Protein disulfide isomerase has also been supplemented to these reactions to yield more active antibodies [62].

##### 3.2.7.2. Large Proteins

The CFPS platform makes the synthesis of very large proteins more tractable in batch mode, allowing for high quantity expression that would normally overwhelm in vivo expression methods [6]. Successful synthesis of soluble, active proteins above 100 kDa has been achieved within the *E. coli*, HeLa, insect, and rabbit reticulocyte platforms. With the high protein producing efficiency of the *E. coli* platform (Figure 2), successful synthesis of the first two (GrsA and GrsB1) of the five modules of a non-ribosomal peptide synthase (NRPS) system was completed, both of which are greater than 120 kDa in size. Specifically, these large proteins were synthesized in batch reactions that ran for 20 h and generated yields of full-length, soluble GrsA at ~106 µg/mL and GrsB1 at ~77 µg/mL [126]. HeLa cell-based CFPS platforms have also demonstrated the ability to synthesize large proteins ranging from 160 to 260 kDa. Namely, this platform produced the proteins GCN2 (160 kDa), Dicer (200 kDa), and mTOR (260 kDa) that were functionally validated with the appropriate biochemical assays [127]. B-galactosidase (116 kDa) was successfully synthesized within an insect platform [18]. The rabbit reticulocyte platform has proved to successfully synthesize active kDa proteins >100, such as a cystic fibrosis transmembrane conductance regulator of ~160 kDa [128].

##### 3.2.7.3. Ice Structuring Proteins

Ice structuring proteins, or antifreeze proteins, are more niche, but still difficult-to-synthesize proteins that benefit greatly from CFPS. These proteins lack common structural features as a family, are difficult to express in whole cells, and require validation of protein products to ensure the active form is successfully produced. CFPS offers more rapid screening and production of both natural and engineered active ice structuring proteins. Ice structuring proteins have been produced successfully in both insect and *E. coli* platforms, and their activity can be tested without the need for purification through an ice recrystallization inhibition assay [129].

##### 3.2.7.4. Metalloproteins

Metalloproteins, such as [FeFe] hydrogenases and multicopper oxidases (MCOs), are difficult to produce in vivo due to low yields, insolubility, poor metal cofactor assembly, and oxygen sensitivity [130,131]. However, they have the potential to enable renewable hydrogen fuel and other important biotechnological advancements. CFPS in the *E. coli* platform has enabled the manipulation of reaction conditions with chemical additives for the synthesis of soluble, active metalloproteins. Specifically, the use of post-CFPS CuSO4 addition for MCO production and the addition of maturation enzymes, iron, and sulfur for [FeFe] reductases greatly improved active enzyme yields [130,131]. Additionally, anaerobic growth of the extract source culture and anaerobic extract preparation were necessary to produce active [FeFe] reductases [131]. The H-cluster of [FeFe] hydrogenase has also been synthesized in *E. coli* CFPS through recreation of the biosynthetic pathway and used to convert apo [FeFe] hydrogenase to active protein [132].

#### 3.2.8. Miscellaneous Applications

CFPS has also been used for a number of miscellaneous applications, including studies of translational machinery, genetic circuits, metabolic engineering, and genetic code expansion. Many of these applications are more feasible and can be used more rapidly in cell-free platforms than in vivo due to the open nature of the system, allowing for faster design–build–test cycles and direct manipulation of the reaction (Figure 1).

##### 3.2.8.1. Studies of Translational Machinery

The open nature of CFPS and the lack of dependence on living cells enables the user to study translational machinery in ways not possible in vivo. These include ribosomal labeling, mutation of ribosomes, removal or replacement of some tRNAs, and generation of orthogonal translation systems, which can improve our understanding of the process of translation across species and help to enable a wider variety of genetic code expansion options [6,133,134]. One such study piloted hybrid ribosome platforms, by supplementing rabbit reticulocyte lysate with other mammalian ribosomes, to prevent energy consumption not directed toward protein synthesis and to boost overall yields [135]. Another study synthesized fully functional ribosomes via the integrated synthesis, assembly, and translation (iSAT) platform [136]. This was achieved through in vitro rRNA synthesis and assembly of ribosomes with supplemented *E. coli* ribosomal proteins. Functionality of these ribosomes was demonstrated by the synthesis of active protein within a single CFPS reaction [136].

##### 3.2.8.2. Genetic Circuits

The challenge for researchers to understand the complexity of gene elements and their interplay in an expedient manner is an ongoing task. Using CFPS for modeling such genetic circuits to further understanding of the dynamics of genetic elements and to program cells capable of executing logical functions provides numerous advantages over in vivo approaches. These include (1) the control of gene and polymerase concentrations, (2) quantitative and rapid reporter measurements, and (3) a larger parameter space that can be evaluated in a high-throughput fashion [137,138]. *E. coli*, wheat germ, and yeast platforms have all exhibited utility in modeling genetic circuits, with *E. coli* extracts being the most widely used. Specifically, *E. coli* and wheat germ extracts have both modeled one-, two-, and three-stage expression cascades within a genetic circuit assembly [139]. *E coli* and yeast extracts have been used as genetic circuits to study the translational noise within cells, determine kinetic parameters, and yield insights within the construction of synthetic genetic networks [140]. Other *E. coli* genetic circuit studies have confirmed and isolated cross talking events, derived a coarse-grained enzymatic description of biosynthesis and degradation, and revealed the importance of a global mRNA turnover rate and passive competition-induced transcriptional regulation among many other studies [141,142,143,144,145,146,147].

##### 3.2.8.3. Metabolic Engineering

The industrial demand for rapid development and screening of commodity chemicals and natural products has prompted the adaptation of CFPS platforms for cell-free metabolic engineering (CFME). This approach allows for a cost-effective platform to produce large amounts of diverse products in a short amount of time [148]. Specifically, CFME provides an in vitro platform comprised of catalytic proteins expressed as purified enzymes or crude lysates that are capable of being mixed to recapitulate full metabolic pathways [148]. The swift prototyping of this approach has already been employed to generate a number of diverse products using yeast and *E.coli*-based platforms [149].

The power of this approach has been used for the production of bio-ethanol using a yeast-based platform to circumvent the limitations of the conventional fermentation process. By employing a bead-beating method to generate yeast cell extract, the CFPS platform was able to generate 3.37 g/L of bio-ethanol compared to 4.46 g/L from the fermentation process at 30 °C. However, the CFPS platform excelled over the fermentation platform at higher temperatures [150]. *E. coli*-based CFME has been optimized for the metabolic conversion of glucose to 2,3-butanediol (2,3-BD) through the engineering of an *E. coli*-based extract to (1) express the genes necessary to convert pyruvate to 2,3-BD, (2) activate cell-free metabolism from glucose, and (3) optimize substrate conditions for highly productive cell-free bioconversions [151]. Additionally, *E. coli* CFME has successfully produced a high titer of mevalonate through systematic production of the enzymes involved in the mevalonate enzymatic pathway and combinatorial mixing of the lysates along with the necessary substrates to recapitulate the full mevalonate enzyme pathway in a biosynthetic manner [148]. Lastly, large NRPS proteins produced in *E. coli* CFPS underwent identical crude lysate mixing approaches to validate their functionality in a metabolic pathway and successfully produced a diketopiperazine in a 12 mg/L concentration [126].

##### 3.2.8.4. Genetic Code Expansion

Genetic code expansion allows for site-specific incorporation of non-standard amino acids (nsAAs) into the protein of interest through reassignment of a codon. This is most commonly achieved through stop codon suppression but can also be done through sense codon reassignment, frameshift codons, or tRNA misacylation [152]. Co-production of an orthogonal tRNA in CFPS has also allowed for nsAA incorporation [153]. Applications of genetic code expansion include incorporation of biophysical probes for structural analysis by NMR, MS, and more, incorporation of fluorophores for interrogation of local protein structures, protein conjugation for production of biomaterials or protein immobilization, incorporation of post-translational modifications, and usage of photocaged amino acids for control of protein activity [152]. While genetic code expansion is possible in vivo, it requires high concentrations of often expensive nsAAs in order to increase the intracellular concentrations to levels high enough for faithful incorporation. The elimination of the cellular barrier in CFPS allows much lower concentrations of nsAA to be used, which can drastically reduce costs (Figure 1) [6,152].

Cell-free genetic code expansion has been accomplished in *E. coli,* insect, rabbit reticulocyte, and wheat germ platforms. The most extensive variety of nsAA incorporations, from hydroxytryptophan to glycosylated serine, has been achieved in *E. coli* [152]. The *E. coli* genome has even been recoded to lack the RF1 gene, and was then capable of 40 incorporations of p-acetyl phenylalanine into an elastin-like polypeptide with 98% accuracy and a 96 µg/mL yield or a single incorporation into GFP with a yield of 550 µg/mL [31,154]. Moreover, suppression of two different stop codons, enabling the incorporation of two different nsAAs into a single protein was achieved in vitro in this platform [155]. One-pot protein immobilization reactions have also been constructed in *E. coli* CFPS reactions, and are achieved using a combination of metal coordination, covalent interactions, or copper-free click chemistry between the protein and activated agarose, glass slides, beads, or silica nanoparticles [156]. This platform has even been used for screening of new aminoacyl tRNA synthetases with adjusted substrate specificity to improve incorporation of new nsAAs [119]. Furthermore*,* methylated oligonucleotides were utilized to sequester tRNAs in active cell extract, allowing for sense codon reassignment directly in the CFPS reaction. The oligo targets a sequence located between the anticodon and variable loop of the tRNA, and is both generic for tRNA type and species, allowing for one-pot sense codon reassignment in multiple cell-free platforms [157]. Additionally, reactions utilizing the expanded genetic code have been prepared by adding purified aminoacyl-tRNA synthetases and an orthogonal-tRNA template directly to the reaction to prevent the need for unique extract preparations for different nsAA incorporations [158].

CFPS allows for rapid screening of nsAA incorporation sites that can affect proper protein folding and yields. Insect CFPS has been used to incorporate p-azido phenylalanine, which was subsequently labeled with a fluorophore, for rapid screening of candidate incorporation sites [152,159,160]. A variety of other nsAAs have also been incorporated in insect, rabbit reticulocyte, and wheat germ platforms. A more in-depth list of many nsAAs that have been incorporated in each platform can be found in “Cotranslational Incorporation of Non-Standard Amino Acids using Cell-Free Protein Synthesis” [152].

### 3.3. Low Adoption Platforms

Cell-free platforms that have experienced low adoption thus far include those derived from *Neurospora crassa*, *Streptomyces*, *Vibrio natriegens*, *Bacillus subtilis*, tobacco, *Arabidopsis*, *Pseudomonas putida*, *Bacillus megaterium*, Archaea, and *Leishmania tarentolae* (Figure 5). These platforms were characterized as low adoption platforms because less than 25 papers have been published for each (Figure 5B and Supplementry Materials). This section will cover both platforms that were created years ago but have only been used for specialized or limited applications, newly emerging platforms, and platforms that are experiencing a revival after years with minimal usage. These platforms are generally less well optimized and well-understood than those covered in the high adoption section, but may still be of interest for certain applications or for further development. We have organized the following based upon platform rather than application to give the reader an overview of the landscape of applications that have been achieved in each platform. For platforms that have not yet had published applications, proposed applications are discussed.

#### 3.3.1. *Neurospora crassa*

A platform utilizing *Neurospora crassa* was created with interest in developing it as a platform for which many gene deletion mutants exist [161]. This was proposed as a way to better study translational quality control utilizing the mutant strains available. This platform has been used to characterize the importance of 7-methylguanosine caps, determine locations of ribosome binding sites, investigate the importance of heat shocking cell cultures and prepared mRNA templates, determine kinetics of luciferase synthesis, and incorporate fluorescent nsAAs to investigate ribosomal stalling [162,163,164,165,166,167].

#### 3.3.2. *Streptomyces*

*Streptomyces* was first used in the 1980s for coupled reactions to express proteins from both linear and circular recombinant *Streptomyces* plasmids, but the original platform fell out of use in the 1990s, likely due to the time-consuming preparation and low yields of the platform [168,169]. Recently, the *Streptomyces* platform has been revived with simplified extract preparation and some improvements to protein yield [15,168]. The platform was optimized with the intention of use for expressing high GC content templates to enable production of natural gene clusters in vitro. With new genome mining technologies, knowledge of natural product gene clusters is increasing rapidly. However, in vivo expression of these clusters results in very low soluble yields due to the high metabolic burden on cells [168]. *Streptomyces-*based CFPS not only accounts for codon optimization for higher GC content templates, but also presents an opportunity for improving soluble expression of natural product gene clusters [15,168]. Examples of high GC content gene expression include *tbrP*, *tbrQ*, and *tbrN* for nonribosomal peptides synthesis of tambromycin as well as the TEII gene involved in valinomycin synthesis [168]. While the *Streptomyces* platform does significantly improve solubility of these proteins compared to expression in *E. coli* CFPS, it does suffer from diminished yields overall, indicating that further optimization of the platform is necessary [168].

#### 3.3.3. *Vibrio natriegens*

Within the last year, the Jewett, Church, and Siemann-Herzberg laboratories have each separately developed a CFPS platform based upon *Vibrio natriegens* [10,170,171]. With its doubling time being the shortest of all known organisms, its high rate of protein synthesis, and high metabolic efficiency, this platform has potential to be an ideal candidate for CFPS [170]. In addition to its unique doubling time*, Vibrio natriegens* extract preparation requires a stationary phase harvest for the highest translational efficiency in a CFPS platform. Typically, CFPS extracts are harvested in a tight window during the mid-exponential phase to maximize translational efficiency. However, the *Vibrio natriegens* extract allows a great amount of flexibility for the user to “set and forget” the culture for a stationary phase harvest where ribosome production is thought to be lowest among other microorganisms [10].

Another advantage to extract preparation for this platform is its high resistance to damage via over-lysis. Additionally, it is relatively agnostic to lysis buffer resuspension volume. Together, these allow for inexperienced CFPS users to easily generate robust extract [10]. In addition, the *V. natriegens* platform generates a very high volume of extract compared to the standard *E. coli* platform, allowing for 8–12 mL of active lysate per L of culture compared to just 1–3 mL/L for *E. coli* when grown in shake flasks and lysed by sonication [10]. *V. natriegens* extract has even been shown to maintain 100% of activity after one week of storage at room temperature post-lyophilization in the presence of trehalose [10]. Although this platform appears to be promising in terms of flexibility and scale of extract preparation, very few applications have been proposed. Aside from reporter proteins being expressed, the Jewett laboratory has demonstrated the successful synthesis of a series of antimicrobial peptides using this platform [10].

#### 3.3.4. *Bacillus subtilis*

The development of a *Bacillus subtilis* CFPS platform has not been pursued until recently due to requirements of exogenous mRNA addition, protease inhibitors, DNase treatments, and less efficient energy systems, as determined by studies in the 1970s and 1980s. These early studies utilized *B. subtilis* extracts to study various antibiotic resistances, investigate bacterial ribosome and mRNA specificity, and identify plasmid replication control genes [172,173,174]. In the last few years, the Freemont laboratory has developed a standardized workflow that circumvents the limitations of the past *B. subtilis* platform. By using a 3-phosphoglycerate (3-PGA) energy regeneration system, with optimized magnesium and potassium glutamate concentrations based upon the *E. coli* CFPS platform, the Freemont laboratory has created a *Bacillus* WB800N platform capable of expressing 0.8 μM GFPmut3b in a reaction that can last for several hours. More research is needed on this platform to reach expression levels seen within the *E. coli* platform, but the Freemont laboratory has successfully characterized an inducible expression platform that was able to quantify the activity of Renilla luciferase. Proposed applications for this platform include the production of industrial or pharmaceutical proteins and applications in metabolic engineering [16].

#### 3.3.5. Tobacco

Though a relatively undeveloped platform, tobacco does allow for a few specific applications and is one of the few plant-based platforms. In the past decades, various parts of the tobacco plant, such as leaves, terminal buds, and trichomes, have been used to prepare extract [175,176,177,178]. These extracts were then used to elucidate differences between 70S and 80S ribosomes, understand synthesis of indoleacetic acid, diterpene cis-abienol, and cytokinins, study cauliflower mosaic virus transcription, and determine nicotine *N*-demethylase activity [176,177,178,179,180,181]. More recently, tobacco BY-2 cells have emerged as the source of extract. Preparation of up to 100 mL of cell extract from BY-2 suspension cultures is possible for larger scale applications [12]. Moreover, successful tobacco extract preparation requires only 4–5 h, whereas other eukaryotic platforms range from 1–5 days (Table 2) [64]. The BY-2 platform has enabled further investigation into positive strand RNA genomes from plant viruses, through synthesis of tomato bushy stunt virus, tomato mosaic virus, brome mosaic virus, and turnip crinkle virus [182,183]. Replicases formed from viral RNAs in CFPS are able to bind to the microsomal structures contained in the extract, allowing for elucidation of the mechanism of genome replication by these viruses, and for the screening of viral mutations [182,183].

Tobacco extract also enables some post-translational modifications, disulfide bond formation, and membrane protein synthesis. The production of a full size, active glucose oxidase antibody and a transmembrane protein has been achieved in this platform without microsomal addition, showing that the extract does contains active endogenous microsomal units that allow for disulfide bond formation, glycosylation, and co-translational membrane integration [12]. However, the full extent of possible PTMs in tobacco CFPS is not well understood. High-throughput coupled reactions from PCR templates with phosphorothioate-modified oligonucleotides have also been created with tobacco extract [12].

#### 3.3.6. *Arabidopsis*

An *Arabidopsis*-based platform was created in 2011, with the proposed advantage of applying the vast knowledge of *Arabidopsis* genetics in combination with CFPS to study post-transcriptional regulation [184]. However, this platform has seen limited actualization of applications, with brief work done on the degradation of uncapped mRNA in mutant cell lines and some investigation into ribosome stalling [184].

#### 3.3.7. *Pseudomonas putida*

Serving as a model organism and understood well at the biochemical level, the Gram-negative bacterium *Pseudomonas putida* has been well established for laboratory research and industrial production of biofuels, recombinant antibody fragments, and natural products. With this already well-established research framework at hand, the Jewett Laboratory has developed and optimized the *P. putida* CFPS platform capable of synthesizing approximately 200 µg/mL of reporter protein within a 4 h, 15 µL batch reaction. Extract preparation for this platform was previously reported, based on that of the *E. coli* platform with slight modifications. Overall, preparation of *P. putida* is faster and less laborious than the *Streptomyces* platform, and is hypothesized to be useful for prototyping the expression of GC-rich genes with codon usage bias. As another high GC bacteria, *P. putida* may be chosen over the *Streptomyces* platform for its aforementioned ease of extract preparation. Moving forward, this platform may also prove useful in screening gene regulatory elements, as well as closing the gap between in vitro and in vivo prediction [14].

#### 3.3.8. *Bacillus megaterium*

In addition to the *Bacillus subtilis* platform, the Freemont laboratory has also begun to pilot a CFPS platform for *Bacillus megaterium*, a large Gram-positive bacterium with potential biotechnology applications including the production of penicillin G amidase, B-amylases, and vitamin B12. Unlike the well characterized *Bacillus subtilis* bacterium species, *B. megaterium* has remained a relatively uncharacterized microbe due to its low-efficiency and time-consuming protoplast transformation procedure. However, creating a CFPS platform to study *B. megaterium* provides some major advantages over *B. subtilis* due to its (1) stable plasmid maintenance, (2) minimal neutral alkaline protease activity, and (3) ability to metabolize low-cost substrates. Currently, this CFPS platform has been used to prototype genetic elements and has demonstrated a protein titer of about 70 μg/mL [185] (Figure 2).

#### 3.3.9. Archaeal

Various archaeal hyperthermophiles and methanogens have been utilized to generate new CFPS platforms, including Methanobacterium formicicium, Methanosarcina barkeri, Methanococcus vannielii, Thermus Thermophilus, Sulfolobus tokodaii, Sulfolobus solfataricus, and Thermococcus kodakarensis.

The thermophilic organisms *S. solfataricus* and *T. kodakaraensis* have been utilized in CFPS for expression of thermophilic proteins, which can be difficult to synthesize in vivo. Ribosomes isolated in cell extracts from these strains are capable of performing at higher temperatures, allowing CFPS reactions to be run at higher temperatures (75 °C for *S. solfataricus*; 65 °C for *T. kodakaraensis*) for improved folding of thermophilic proteins [20,186]. However, other problems with high-temperature CFPS reactions have yet to be fully mitigated. For example, production of chitinase in *T*. *kodakarensis* CFPS stopped after 30 min, which was conjectured to be an issue with energy depletion worsened by the shorter half-life of energy-rich molecules at high temperatures [20]. Additionally, coupled reactions are not yet feasible at elevated temperatures, due to the differences in optimal performance temperatures for transcription and translation reactions [20].

Many archaeal methanogenic CFPS platforms have also been used to probe antibiotic sensitivity in order to elucidate phylogenetic connections. Antibiotic targeting to ribosomes can be confirmed using CFPS platforms in a way not possible in vivo because cell viability is inconsequential [187]. Antibiotic enhancement of neomycin and paromomycin and the physiological roles of polyamines were also investigated in *T. thermophilus* and *S. tokodaii* CFPS platforms [188,189].

#### 3.3.10. *Leishmania tarentolae*

*Leishmania tarentolae*, a protozoan platform, is a relatively new platform that has experienced some recent optimization. *L. tarentolae* appears to be particularly promising for growth and extract scalability, with a relatively short doubling time and faster extract preparation when compared to eukaryotes of interest [190].

*L. tarentolae-*based CFPS has been utilized for a variety of high-throughput applications, with CFPS possible directly from PCR templates and protein analysis possible directly in the reaction mixture. One type of analysis utilizes fluorescence cross-correlation spectroscopy to analyze protein–protein or protein–small-molecule interactions [11,190]. Protein arrays can also be constructed in time and cost-effective ways in the *L. tarentolae* platform by utilizing “translation and immobilization of protein on hydrophobic substrate” (TIPoHS). Here, CFPS reactions are run on membranes, and immobilization and detection are achieved via a c-terminal GFP tag [191].

The *L. tarentolae* platform has been used for disulfide bond formation, and while other PTMs may be possible, they are not yet well defined or understood [64,192]. The platform is also capable of membrane protein synthesis with the addition of liposomes or nanodiscs, and was used to synthesize 22 different human solute carrier proteins [193]. Along with *E. coli,* methylated oligonucleotides have been used to sequester tRNAs for one-pot sense codon reassignment, allowing for genetic code expansion in *L. tarentolae* [157].

### 3.4. Recent and Future Applications

An incredible diversification of CFPS usage has occurred since its inception in 1961. In the last three years alone, there have been a handful of key new applications that have contributed greatly to the field of CFPS. These include the first instances of CFPS used for education, for the development of one-pot reactions for glycoprotein synthesis, for sense codon reassignment, for protein immobilization, for continued refinement of lyophilization for better shelf stability of cell-free extract, and for the demonstration of multiple non-standard amino acid incorporations into a single protein [43,46,47,53,54,154,155,156,157]. Furthermore, a handful of promising new and revived CFPS platforms from *Streptomyces, Pseudomonas putida*, *Bacillus subtilis*, *Bacillus megaterium*, and *Vibrio natriegens* have been introduced for novel applications, including the synthesis of proteins from high GC templates (*Streptomyces*; *P. putida*), and for the further development of applications such as metabolic engineering (*B. subtilis*) [10,14,15,16,168,170,171].

Despite the proliferation of CFPS platforms and applications in the last 60 years, there are still many new directions in which the technology can be taken. Some future directions for CFPS may include further development and optimization of current platforms, especially the emerging or re-emerging platforms of *Streptomyces*, *Pseudomonas*, *Bacillus subtilis*, *Bacillus megaterium*, and *Vibrio natriegens*. Soon, the proposed applications of these platforms may be actualized. Furthermore, we may see additional CFPS platforms be established to solve new problems or to fill other existing gaps that current platforms have left. In terms of applications, there may be more utilization of CFPS for education, metabolic engineering, personalized medicine, and diagnostics, as current work seems to have only scratched the surface of these applications. Further development of large-scale CFPS may also be a future direction developed alongside these applications in order to support new industrial endeavors.

## 4. Methodological Differences between Platforms

While the user’s selection of a given CFPS platform will be primarily driven by the applications enabled by a platform, there are often multiple platforms that can be used for a single type of application. The choice between these platforms can be guided by factors including the accessibility and technical complexity of the methods used to produce the cell extract, the reagents used for CFPS reactions, the type of reaction (coupled vs uncoupled), and the productivity of the platform. Here we provide further guidance to the user in choosing the platform that best suits their needs, and simplify the effort needed to make this choice by providing a condensed methodological comparison of the high adoption cell-free platforms: *E. coli*, insect, yeast, Chinese hamster ovary, rabbit reticulocyte lysate, wheat germ, and HeLa cells (Table 1, Table 2 and Table 3). 

### 4.1. Productivity

Firstly, different platforms will be better suited for the production of different proteins of interest, and maximizing protein yields is not required for all applications. Therefore, matching the application with a platform’s productivity will enrich success for new users (Figure 2). For example, industrial level protein production is currently best enabled by *E. coli* or wheat germ platforms, with possibilities of large-scale protein production in the emerging *Vibrio natriegens* and *Pseudomonas putida* platforms (Figure 4 and Figure 5). However, for many applications that may not require large protein samples, such as small-scale assays or functional investigations, most possible platforms would still provide large enough yields. In general, eukaryotic platforms give lower protein yields, with the exception of the wheat germ platform (Figure 2). On the lowest end of the productivity scale are the rabbit reticulocyte and archaeal platforms, which produce under 20 µg/mL of protein in batch format (Figure 2). Overall, it is important to choose a platform that is suited to producing the protein of interest in the quantity necessary for the desired application.

### 4.2. Growth

Methodology for cell growth from representative sources for each high adoption platform is summarized in Table 1. Growth media is highly variable between platforms, as would be expected even in in vivo protein expression. Additionally, cells can be grown in a variety of vessels, from baffled flasks in an incubator for wheat germ and *E. coli* to fermenters and spinner flasks for insect, Chinese hamster ovary*,* and HeLa cells. The vessel choice may also depend on the growth scale desired. Lastly, cell cultures must be harvested, which is typically done via centrifugation and washing of the pelleted cells. Platforms that stand out most due to specialized methods are wheat germ and rabbit reticulocyte. In general, all other platforms utilize cell growth in liquid culture, centrifugation for the harvest of cells, and pellet washing in an HEPES-based buffer supplemented with acetate salts and with DTT in some cases. However, for wheat germ, wheat seeds are ground in a mill and sieved, and embryos are selected by solvent flotation [194]. Rabbit reticulocyte extract preparation may even require treatment of live rabbits to make them anemic as well as bleeding of the rabbits to obtain the cells needed [98].

### 4.3. Extract Preparation

Extract preparation consists of pre-lysis preparation, lysis, and post-lysis processing, which are covered in detail for each high adoption platform in Table 2. Lysis methods not only vary from platform to platform, but many different lysis methods can also be used for a single platform. Here we have highlighted just one of the methods used for each platform, but others may also be viable. Firstly, cells are resuspended, then sonication (*E. coli*, wheat germ), homogenization (yeast), nitrogen cavitation (HeLa, insect), freeze-thaw (insect), syringing (Chinese hamster ovary), osmotic lysis (rabbit reticulocyte), or other lysis methods may be used to disrupt cell membranes. The lysate is centrifuged at high speeds to separate out cell membrane fragments and other unnecessary cellular debris. Post-processing after lysis and centrifugation also varies from platform to platform. For example, a run-off reaction, where the supernatant is incubated, is performed on *E. coli* extract. For Chinese hamster ovary, HeLa, insect, wheat germ, and yeast, desalting or dialysis is performed on the supernatant. The Chinese hamster ovary, HeLa, and rabbit platforms are generally treated with micrococcal nuclease to degrade remaining endogenous mRNA in the extract, and the nuclease activity is quenched through addition of EGTA. All extracts are then flash frozen in liquid nitrogen and stored either in liquid nitrogen, or more frequently at −80 °C if CFPS is not immediately performed afterwards.

### 4.4. CFPS Reaction Setup

CFPS reaction setup requires mixing of many reagents to initiate protein synthesis, and the details of setup for each high adoption platform are covered in Table 3. There are two main differences among CFPS setups: the chosen energy system and whether the reaction is coupled or uncoupled. Otherwise, the reaction components are generally the same, with two unique reagents used for each platform and slight variations in concentration from platform to platform. Common reagents include ATP, GTP, UTP, CTP, tRNA, HEPES, Mg salts, K salts, 20 amino acids, and energy rich molecules. Most platforms use a creatine phosphate/creatine kinase energy system, and the most work has been done in *E. coli* to enable more inexpensive energy systems, such as PEP, glucose, and maltodextrin [8,197]. Reaction temperature has also been a major point of optimization for each of these platforms, with typical temperatures ranging from 21 to 37 °C among the various platforms [17,195,198] (Table 2). In terms of reaction type, coupled reactions are desirable because of the ease of setup, but uncoupled reactions are typically used for eukaryotic platforms to improve yields (see Section 2.2, titled “Coupled and Uncoupled Formats”) [28]. Uncoupled reactions require an in vitro transcription reaction often catalyzed by T7 RNA polymerase (T7RNAP), followed by mRNA purification, then a cell-free translation reaction utilizing the prepared lysate, and are both more time-consuming and more difficult in terms of handling. Platforms that generally utilize uncoupled reactions include wheat germ, rabbit reticulocyte, insect, and HeLa. Transcription for most platforms that utilize coupled reactions require T7RNAP, but some platforms, such as *E. coli* are able to employ solely the endogenous polymerase [199,200].

### 4.5. Time

Overall, wheat germ and rabbit reticulocyte are the most time-consuming preparations, at 4–5 days for wheat germ and up to 9 days for rabbit, if treatment of animals is needed. All other platforms hover around the 1–2 day mark for preparation, with highly variable growth times dependent on doubling time for the strain and final cell density desired for harvest. *E. coli* requires the least time for preparation from the initiation of culture growth to the final freezing of extract due to its quick doubling time and relatively simple extract preparation procedure.

## 5. Standard Optimizations

A variety of internal development of the CFPS platforms is constantly occurring in order to improve protein yields and streamline extract preparation. Some major advances have greatly improved a variety of the CFPS platforms, such as internal ribosome entry sites (IRESs), species-independent translational leaders (SITS), and 5′UTR optimization. These have improved the rates of translation in eukaryotic platforms, which can limit protein yield. 5′UTRs are used to mimic cap structures and promote binding of the ribosome to the mRNA template, but in some cases they have also been found to be unhelpful or even detrimental to productivity. Additionally, 5′UTR choice may require some testing and optimization before application [6,11,19,202]. IRESs are sequences utilized by viruses to hijack cellular machinery for replication. They have been added to CFPS templates in order to bypass translation initiation factors, but many are species-dependent. However, IRESs have been used in rabbit reticulocyte, Chinese hamster ovary, yeast, and *Leishmania tarentolae* [64,203,204,205]. SITS are unstructured translation leaders that allow transcribed mRNA to interact directly with ribosomes across a variety of CFPS platforms from many cell types, such that translation initiation factors are not needed [11,190,193]. Codon optimization of the template DNA has also been used to improve yields in eukaryotic platforms [73].

In addition to template optimization, many high adoption platforms have undergone optimization of cell-free reaction reagent concentrations through systematic titrations of the main reagents [197,206]. Additionally, protein yields can be augmented by the addition of purified transcriptional and translational components or molecular crowding agents [207,208].

## 6. Conclusions

This review is aimed at helping new users of CFPS platforms determine which platform best suits their needs. We sought to highlight similarities and differences among the platforms, the applications that can be achieved by each, and the reasons one platform may be more advantageous for a certain goal than another.

We recommend new users first investigate the high adoption platforms to find one that suits them, as these platforms have been best optimized and there is plentiful literature to support the user. High adoption platforms include *E. coli*, insect, yeast, Chinese hamster ovary, rabbit reticulocyte, wheat germ, and HeLa. For these platforms, we have covered a wide spectrum of applications that are enabled by each, to provide the reader with an idea of the breadth of possibilities in CFPS, as well as to hopefully cover a wide spectrum of user needs. These applications include education, post-translational modifications, high-throughput expression, virus-like particles production, large-scale synthesis, membrane proteins, difficult-to-synthesize proteins (antibodies, large proteins, ice-structuring proteins, and metalloproteins), and miscellaneous applications (studies of translational machinery, genetic code expansion, metabolic engineering, and genetic circuits). In addition, we have covered the methods for growth, extract preparation, and cell-free reaction setup, as well as batch reaction protein yield, such that the reader can further determine if the platform suits their needs and obtain a better understanding of what is required for successful implementation of each.

We also briefly covered the applications enabled by low adoption platforms including Neurospora crassa, Streptomyces, Vibrio natriegens, Bacillus subtilis, tobacco, Arabidopsis, Pseudomonas putida, Bacillus megaterium, Archaea, and Leishmania tarentolae. While these platforms have some work supporting their use, they have generally been used by only a few and are not as well optimized. However, these platforms may still provide some key advantages to the field if more work is done with them. Additionally, the emerging platforms of Vibrio natriegens, Streptomyces, Bacillus subtilis, Bacillus megaterium, and Pseudomonas putida are proposed to enable exciting new applications of CFPS, including natural product synthesis from high GC templates.

## Figures and Tables

**Table 1:** Comparison of growth methods for high adoption platforms. We have assembled the major growth methodologies for each of the high adoption platforms to give users an idea of the relative differences between them. These are not the only techniques that have been used for growth for each platform, but they are generally representative of the methods.

Growth			
Platform	Media/Vessel	Harvest	Key Citations
***E. coli***	**Media:** 2× YPTG (5 g NaCl, 16 g Tryptone, 10 g Yeast extract, 7 g KH_2_PO_4_, 3 g KHPO_4_, pH 7.2/750 mL solution, 18 g Glucose/250 mL solution). **Vessel:** 2 L Baffled Flask. **Conditions:** 37 °C, 200 RPM	When OD_600_ is 3, centrifuge at 5000× *g* for 10 min at 10 °C. Wash pellet with 30 mL S30 buffer (10 mM Tris OAc, pH 8.2, 14 mM Mg(OAc)_2_, 60 mM KOAc, 2 mM DTT), then centrifuge at 5000× *g* for 10 min at 10 °C. Repeat wash 3 times in total.	[22]
**Wheat Germ**	Grind wheat seeds in a mill.	Sieve through 710–850 mm mesh, select embryos via solvent flotation method using a solvent containing 240:600 *v*/*v* cyclohexane and carbon tetrachloride. Dry in fume hood overnight.	[194]
**Yeast**	**Media:** 2% *w*/*v* Peptone, 1% *w*/*v* Yeast extract, 2% *w*/*v* Glucose **Vessel:** 2.5 L baffled flask **Conditions:** 30 °C, 250 RPM	When OD_600_ of 10–12 is reached, centrifuge culture for 10 min at 3000× *g*. Wash pellet with Buffer A (20 mM HEPES, pH 7.4, 100 mM KOAc, 2 mM Mg(OAc)_2_). Centrifuge for 10 min at 3000× *g* and 4 °C. Repeat washing 3 times.	[19]
**Rabbit Reticulocyte**	Make rabbits anemic over 3 days by injections of APH.	Bleed rabbits on day 8. Filter blood through cheesecloth and keep on ice, then centrifuge at 2000 RPM for 10 min.	[98]
**Insect**	**Media:** Animal component free insect cell medium. **Vessel:** Fermentor. **Conditions:** 27 °C	When cell density reaches 4 × 10^6^ cell/mL, centrifuge culture at 200× *g* for 10 min. Wash once with buffer (40 mM HEPES KOH, pH 7.5, 100 mM KOAc, 4 mM DTT).	[129]
**HeLa**	**Media:** Minimal essential medium supplemented with 10% heat-inactivated fetal calf serum, 2 mM glutamine, 1 U/mL penicillin, 0.1 mg/mL streptomycin. **Vessel:** Spinner flask with cell culture controller **Conditions:** 37 °C, pH 7.2, 67 ppm oxygen, 50 RPM	Harvest when cell density reaches 0.7–0.8 × 10^6^ cells/mL. Wash 3 times with buffer (35 mM HEPES KOH, pH 7.5, 140 mM NaCl, 11 mM glucose).	[13]
**Chinese Hamster Ovary**	**Media:** Power Chinese hamster ovary-2 chemically defined serum-free media **Vessel:** Fermentor **Conditions:** 37 °C	Harvest at 2 × 10^6^ cells/mL cell density by centrifuging at 200× *g* for 10 min. Wash cells once with buffer (40 mM HEPES KOH, pH 7.5, 100 mM NaOAc, 4 mM DTT).	[17]

**Table 2:** Comparison of extract preparation methods for high adoption platforms. We have assembled the major extract preparation methodology for each of the high adoption platforms to give users an idea of the relative differences between them. These are not the only techniques that have been used for extract preparation for each platform, but they are generally representative of the methods.

Extract Prep					
Platform	Pre-Lysis	Lysis	Post-Lysis Processing	Growth and Prep Time	Key Citations
***E. coli***	Resuspend in 1 mL/1 g pellet of S30 buffer by vortexing.	Sonicate on ice for 3 cycles of 45 s on, 59 s off at 50% amplitude. Deliver 800–900 J total for 1.4 mL of resuspended pellet. Supplement with a final concentration of 3 mM DTT.	Centrifuge lysate at 18,000× *g* and 4 °C for 10 min. Transfer supernatant while avoiding pellet. Perform runoff reaction on supernatant at 37 °C and 250 RPM for 60 min. Centrifuge at 10,000× *g* and 4 °C for 10 min. Flash freeze supernatant and store at −80 °C.	1–2 days	[22]
**Wheat Germ**	Wash 3 times with water under vigorous stirring to remove endosperm.	Sonicate for 3 min in 0.5% Nonidet P-40. Wash with sterile water. Grind washed embryos into fine powder in liquid nitrogen and resuspend 5 g in 5mL of 2× Buffer A (40 mM HEPES, pH 7.6, 100 mM KOAc, 5 mM Mg(OAc)_2_, 2 mM CaCl, 4 mM DTT, 0.3 mM of each of the 20 amino acids).	Centrifuge at 30,000× *g* for 30 min. Filter supernatant through G-25 column equilibrated with Buffer A. Centrifuge column product at 30,000× *g* for 10 min. Adjust to 200 A_260_/mL with Buffer A. Store in liquid nitrogen.	4–5 days	[64,194]
**Yeast**	Resuspend cells in 1 mL lysis buffer (20 mM HEPES KOH, pH 7.4, 100 mM KOAc, 2 mM Mg(OAc)2, 2 mM DTT, 0.5 mM PMSF) per 1 g cell pellet.	Pass through homogenizer once at 30,000 psig.	Centrifuge homogenate at 30,000× *g* for 30 min at 4 °C. Then repeat centrifugation with supernatant in a spherical bottom bottle. Desalt supernatant in dialysis tubing with 4 exchanges of 50× volume lysis buffer for 30 min each at 4 °C. Centrifuge at 60,000× g for 20 min at 4 °C. Flash freeze and store at −80 °C.	1–2 days	[19,195]
**Rabbit Reticulocyte**	Resuspend cells in buffered saline with 5 mM glucose, then centrifuge at 2000 RPM for 10 min. Repeat wash 3 times.	Resuspend cells in 1.5 times the packed cell volume of ice-cold water, mix thoroughly.	Spin lysate at 15,000× *g* for 20 min at 2 °C. Pour supernatant though 53 μm nylon. Treat with micrococcal nuclease by adding 0.2 mL of 1 mM hemin, 0.1 mL of 5 mg/mL creatine kinase, 0.1 mL of 0.1 M CaCl_2_, 0.1 mL of micrococcal nuclease. Incubate at 20 °C for 15 min, then add 0.1 mL of 0.2 M EGTA and 60 μL of 10 mg/mL tRNA. Store in liquid nitrogen or at −70 °C.	~8 days to treat rabbits, 1 day for extract preparation	[98]
**Insect**	Resuspend cells in buffer to final density of 2 × 10^8^ cells/mL.	Mechanically lyse cells by rapidly freezing in liquid nitrogen, then thawing in water bath at 4 °C or use a disruption chamber with 20 kg/cm^2^ nitrogen gas for 30 min.	Centrifuge lysate at 10,000× *g* for 10 min. Apply supernatant to G-25 gel filtration column. Pool fractions with highest A_260_, flash freeze in liquid nitrogen and store at −80 °C.	1–2 days	[18,129,195,196]
**HeLa**	Resuspend in extraction buffer (20 mM HEPES KOH, pH 7.5, 135 mM KOAc, 30 mM KCl, 1.655 mM Mg(OAc)_2_) to ~2.3 × 10^8^ cells/mL.	Disrupt cells via 1 MPa nitrogen pressure for 30 min in a cell disruption chamber.	Centrifuge homogenate at 10,000× *g* for 5 min at 4 °C. Pass supernatant through a PD-10 desalting column equilibrated with extraction buffer. Treat 100 μL of extract with 1 μL of 7500 U/mL nuclease S7 and 1 μL of 100 mM CaCl_2_ for 5 min at 23 °C, then add 8 μL of 30 mM EGTA. Flash freeze eluted extract in liquid nitrogen and store at −80 °C.	1–2 days	[13,195]
**Chinese Hamster Ovary**	Resuspend cells in buffer to density of 5 × 10^8^ cells/mL.	Disrupt cells by syringing the pellet through a 20-gauge needle.	Centrifuge lysate at 10,000× *g* for 10 min. Apply supernatant to G-25 gel filtration column. Pool fractions with an A_260_ above 100. Treat pooled fractions with 10 U/mL S7 nuclease and 1 mM CaCl_2_, incubate at room temperature for 2 min, then add 6.7 mM EGTA. Flash freeze in liquid nitrogen and store at −80 °C.	1–2 days	[17]

**Table 3:** Comparison of cell-free protein synthesis reaction setup for high adoption platforms. This table is intended to help users understand major differences between setups for various high adoption platforms, namely whether reactions are generally coupled or uncoupled, what energy systems are typical, and what temperatures the reactions are run at. These are not the only setups that have been used for successful cell-free protein expression in each platform, but they are generally representative of the reagents, concentrations, and temperatures used for each platform.

Cell-Free Protein Synthesis Reaction				
Platform	Vessel/Conditions	Reaction Composition	Energy Systems	Key Citations
***E. coli***	**Vessel:** Many vessels can be used, yield increases as the surface area to reaction volume ratio increases **Conditions:** 30 °C overnight or 37 °C for 4 h	33% *v*/*v E. coli* extract, 16 μg/mL T7RNAP, 16 ng/mL DNA template, Solution A (1.2 mM ATP, 0.85 mM GTP, 0.85 mM UTP, 0.85 mM CTP, 31.50 μg/mL Folinic Acid, 170.60 μg/mL tRNA, 0.40 mM Nicotinamide Adenine Dinucleotide (NAD), 0.27 mM Coenzyme A (CoA), 4 mM Oxalic Acid, 1 mM Putrescine, 1.50 mM Spermidine, 57.33 mM HEPES buffer), Solution B (10 mM Mg(Glu)_2_, 10 mM NH_4_(Glu), 130 mM K(Glu), 2 mM of each amino acid, 0.03 M Phosphoenolpyruvate (PEP))	PEP, glucose + glutamate decarboxylase, or maltodextrin are possible	[22,201]
**Wheat Germ**	**Vessel:** Not noted **Conditions:** 26 °C	First, perform an in vitro transcription reaction and isolate mRNA using SP6 RNA polymerase. Set up cell-free translation as follows: 24% *v*/*v* wheat germ extract, 4 mM HEPES KOH, pH 7.8, 1.2 mM ATP, 0.25 mM GTP, 16 mM creatine phosphate, 0.45 mg/mL creatine kinase, 2 mM DTT, 0.4 mM spermidine, 0.3 mM of each of the 20 amino acids, 2.5 mM Mg(OAc)_2_, 100 mM KOAc, 50 μg/mL deacylated tRNA from wheat embryos, 0.05% Nonidet P-40, 1 μM E-64 as proteinase inhibitor, 0.005% NaN_3_, 0.02 nmol mRNA.	Creatine phosphate + creatine kinase	[194]
**Yeast**	**Vessel:** 15 μL reactions in 1.5 mL microfuge tubes **Conditions:** 21 °C	25 mM HEPES KOH, pH 7.4, 120 mM K(Glu), 6 mM Mg(Glu)_2_, 1.5 mM ATP, 2 mM GTP, 2 mM CTP, 2 mM UTP, 0.1 mM of each of 20 amino acids, 25 mM creatine phosphate, 2 mM DTT, 0.27 mg/mL creatine phosphokinase, 200 U/mL RNase Inhibitor, 27 μg/mL T7 RNAP, DNA template, and 50% *v*/*v* yeast extract	Creatine phosphate + creatine kinase	[19,195]
**Rabbit Reticulocyte**	**Vessel:** 200 μL reaction performed in an NMR spectrometer **Conditions:** 30 °C	First, perform an in vitro transcription reaction and isolate mRNA using T7 RNAP. Supplement 1 mL of rabbit reticulocyte lysate with 25 μM hemin, 25 μg creatine kinase, 5 mg phosphocreatine, 50 μg of bovine liver tRNAs, and 2 mM D-glucose. Initiate in vitro translation by combining 27 nM of in vitro transcribed mRNAs, 50% *v*/*v* supplemented lysate, 75 mM KCl, 0.75 mM MgCl_2_, and 20 μM amino acids mix.	Creatine phosphate + creatine kinase	[135]
**Insect**	**Vessel:** 25 μL reaction, vessel size not noted **Conditions:** 25 °C	First, perform an in vitro transcription reaction and isolate mRNA using T7 RNAP. Then set up cell-free translation as follows: 1.5 mM Mg(OAc)_2_, 0.25 mM ATP, 0.1 mM GTP, 0.1 mM EGTA, 40 mM HEPES KOH, pH 7.9, 100 mM KOAc, 20 mM creatine phosphate, 200 μg/mL creatine kinase, 2 mM DTT, 80 μM of each of the 20 amino acids, 0.5 mM PMSF, 1 U/µL RNase inhibitor, 200 μg/mL tRNA, 320 μg/mL mRNA, and 50% *v*/*v* insect cell extract. Addition of 20% *v*/*v* glycerol to the reaction was also shown to improve yields.	Creatine phosphate + creatine kinase	[18]
**HeLa**	**Vessel:** 6 μL reaction, vessel not noted **Conditions:** 32 °C, 1 h	First, perform an in vitro transcription reaction and isolate mRNA using T7 RNAP. Cell-free translation is performed as follows: 75% *v*/*v* HeLa cell extract, 30 μM of each of the 20 amino acids, 27 mM HEPES KOH, pH 7.5, 1.2 mM ATP, 0.12 mM GTP, 18 mM creatine phosphate, 0.3 mM spermidine, 44–224 mM KOAc, 16 mM KCl, 1.2 mM Mg(OAc)_2_, 90 μg/mL calf liver tRNA, 60 μg/mL creatine kinase, and purified mRNA.	Creatine phosphate + creatine kinase	[13]
**Chinese Hamster Ovary**	**Vessel:** 25 μL reaction, vessel size not noted **Conditions:** 33 °C, 500 RPM shaking in thermomixer	25% *v*/*v* Chinese hamster ovary cell extract, 100 μM of each of the 20 amino acids, 1.75 mM ATP, 0.30 mM CTP, 0.30 mM GTP, 0.30 mM UTP, 20 nM DNA template, 1 U/μL T7 RNAP, 30 mM HEPES KOH, pH 7.6, 150 mM KOAc, 3.9 mM Mg(OAc)_2_, 20 mM creatine phosphate, 100 μg/mL creatine kinase, 0.25 mM spermidine, and 2.5 mM DTT.	Creatine phosphate + creatine kinase	[17]

## Supplementary Materials

The following are available online at https://www.mdpi.com/2409-9279/2/1/24/s1: Spreadsheet 1: PubMed search results for high adoption platforms; Spreadsheet 2: Curated PubMed search results for low adoption platforms.
